# Multidimensional Factors Can Explain the Clinical Worsening in People With Parkinson's Disease During the COVID-19 Pandemic: A Multicenter Cross-Sectional Trial

**DOI:** 10.3389/fneur.2021.708433

**Published:** 2021-07-30

**Authors:** Carla Silva-Batista, Daniel Boari Coelho, Renato Campos Freire Júnior, Lorena Rosa Almeida, Adriana Guimarães, Katia Cirilo Costa Nóbrega, Hugo Machado Sanchez, Ana Raquel Rodrigues Lindquist, Vera Lúcia Israel, Hélcio Kanegusuku, Rachel Guimarães, Nayanne Beckmann Bosaipo, Richelma Barbosa, Clynton Lourenço Correa, Maria José Finatto, Felipe Augusto dos Santos Mendes, Maria Elisa Pimentel Piemonte

**Affiliations:** ^1^Exercise Neuroscience Research Group, University of São Paulo, Sao Paulo, Brazil; ^2^Biomedical Engineering, Federal University of ABC, Sao Bernardo do Campo, Brazil; ^3^Faculty of Physical Education and Physical Therapy, Federal University of Amazonas, Manaus, Brazil; ^4^Movement Disorders and Parkinson's Disease Clinic, Roberto Santos General Hospital/SESAB, Salvador, Brazil; ^5^Motor Behavior and Neurorehabilitation Research Group, Bahiana School of Medicine and Public Health, Salvador, Brazil; ^6^Department of Physical Education, State University of Santa Catarina, Florianópolis, Brazil; ^7^Department of Physiotherapy, Federal University of Amapa, Macapa, Brazil; ^8^Physical Therapy Department, Federal University of Jataí, Jataí, Brazil; ^9^Graduate Program of Physical Therapy, Federal University of Rio Grande do Norte, Natal, Brazil; ^10^Department of Physiotherapy, Federal University of Parana, Curitiba, Brazil; ^11^Graduated Program in Rehabilitation Sciences, Universidade Nove de Julho, Sáo Paulo, Brazil; ^12^Laboratory of Neuroimaging, University of Campinas, Sáo Paulo, Brazil; ^13^Setor de Distúrbios do Movimento e Neurologia Comportamental do Hospital das Clínicas da Faculdade de Medicina de Ribeirão Preto da Universidade de São Paulo, Sáo Paulo, Brazil; ^14^Department of Physiotherapy, University of Pará State, Santarém, Brazil; ^15^Graduate Program of Physical Education, Federal University of Rio de Janeiro, Rio de Janeiro, Brazil; ^16^Department of Linguistics, Federal University of Rio Grande do Sul, Porto Alegre, Brazil; ^17^Graduate Program in Rehabilitation Sciences, Faculty of Ceilandia of University of Brasília, Brasília, Brazil; ^18^Faculty of Medical Science of University of São Paulo, Sáo Paulo, Brazil

**Keywords:** non-motor symptoms, motor symptoms, mental health, social distancing, physical activity

## Abstract

**Background:** Self-reported clinical worsening by people with Parkinson's disease (PD) during social distancing may be aggravated in Brazil, where the e/tele-health system is precarious.

**Objectives:** This study aims to investigate self-reported changes in motor and non-motor aspects during social distancing in people with PD living in Brazil and to investigate the factors that might explain these changes.

**Methods:** In this multicenter cross-sectional trial, 478 people with a diagnosis of idiopathic PD (mean age = 67, *SD* = 9.5; 167 female) were recruited from 14 centers distributed throughout the five geographical regions of Brazil. The evaluators from each center applied a questionnaire by telephone, which included questions (previous and current period of social distancing) about the motor and non-motor experiences of daily living, quality of life, daily routine, and physical activity volume.

**Results:** Self-reported clinical worsening in non-motor and motor aspects of daily life experiences (Movement Disorder Society-Unified PD Rating Scale—parts IB and II—emotional and mental health, and fear of falling) and in the quality of life was observed. Only 31% of the participants reported a guided home-based physical activity with distance supervision. Perceived changes in the quality of life, freezing of gait, decreased physical activity volume, daily routine, and fear of falling explained the self-reported clinical worsening (*P* < 0.05).

**Conclusions:** Self-reported clinical worsening in people with PD living in Brazil during social distancing can also be aggravated by the precarious e/tele-health system, as perception of decreased physical activity volume and impoverishment in daily routine were some of the explanatory factors. Considering the multifaceted worsening, the implementation of a remote multi-professional support for these people is urgent.

## Introduction

The coronavirus disease 2019 (COVID-19) is an infectious disease of pandemic proportions, which is rapidly developing around the world. A higher COVID-19 mortality rate has been described in people with advanced Parkinson's disease (PD) in association with older age and longer disease duration (1). Attempts to curb COVID-19 have forced countries to be under lockdown, with strict emphasis on self-isolation and social distancing. Despite being effective for infection control, social distancing has negative effects on PD.

People with PD living in Italy reported an acute clinical worsening in either motor disturbances or neuropsychiatric non-motor symptoms due to lockdown and infection outbreak (2), consistent with clinical worsening in mental health and reduced physical activity level in people with PD living in Egypt (3), India (4), and Germany (5). Perceptions of negative changes in daily routine, freezing of gait, fear of falling, and fear of the COVID-19 pandemic have been observed during lockdown in people with PD (1, 4, 6), which are important aspects that may potentially lead to the worsening of motor and non-motor symptoms and mental health in this population. In addition, the motor and non-motor symptoms of people with PD living in Italy significantly worsened in those infected by COVID-19 (7). These studies showed a clinical worsening of PD during the COVID-19 pandemic in a small sample size (≤100 people with PD), although there is no systematic data available (8). However, there is no evidence about the impact of social distancing on the clinical condition of people with PD living in Brazil.

Brazil is a continental country with large socioeconomic differences, where people with PD have to deal with the deficiency of the healthcare system toward disease treatment and with precarious telemedicine and e-health systems (9). In addition, the restricted access to healthcare and physical exercise during social distancing might produce additional stress and worsening of motor and non-motor symptoms in this population (10), which have been reported by people with PD living in Egypt during the COVID-19 lockdown (3). Although the lockdown has not been implemented in Brazil, the recommendation for social distancing has remained for longer than 2 months, which might enhance the self-reported clinical worsening in people with PD living in Brazil.

Therefore, this study investigated the self-reported clinical aspects (motor and non-motor aspects of daily life experiences and emotional and mental health) of people with PD living in Brazil during social distancing due to the COVID-19 pandemic. We also investigated the factors that might explain the self-reported clinical aspects (motor and non-motor aspects of daily life experiences and emotional and mental health) of PD during social distancing.

## Materials and Methods

### Study Design and Participants

This multicenter cross-sectional trial was conducted between May and June 2020. The Brazilian Ministry of Health confirmed the first case of COVID-19 in February 25, 2020; as no lockdown was imposed, social distancing (e.g., physically distancing from other people and staying at home or avoiding crowded areas) was initiated on March 11, 2020. People with a confirmed diagnosis of idiopathic PD according to the diagnostic criteria of the UK Parkinson's Disease Society Brain Bank (11) who are users of health services in the 14 centers distributed in the five geographical regions of Brazil (South, Southeast, Midwest, Northeast, and North) were recruited to participate in this study. The eligibility criteria were (a) ≥40 years old and (b) being treated for PD in the 6 months preceding the commencement of the study. The non-eligibility criteria were (a) the presence of neurological disorders other than PD and (b) the presence of significant cognitive, speech, and hearing disorders since interviews were conducted by phone calls or phone messages. To assess the cognitive status, we considered the ability of the participants to properly answer the first section of the study questionnaire about personal and socio-economic information as clinical evidence about the minimal cognitive capacity to self-evaluate their health condition. We confirmed their answers with the answers of the caregivers.

It is important to highlight that the Hoehn and Yahr stage was defined based on the responses of the participants about four crucial points: (1) if the PD symptoms had started in one side of the body, (2) if the symptoms had progressed to both sides of the body, (3) if balance was impaired due to disease progression, and (4) if the participants were able to walk/stand up independently.

### Recruitment

We contacted (up to two attempts on two different days) the eligible patients according to the data of the centers. After being informed about the procedures of the study, the patients were asked to give their consent to participate. This study was approved by the Ethics Committee of the General Hospital of Faculty of Medicine of the University of São Paulo (#CAAE 67388816.2.0000.0065) and conducted in accordance with the Helsinki Declaration.

### Sample Size

A convenience sample (478 people with PD) was used in the present study because there is no systematic data available for people with PD during social distancing (8).

### Study Procedures

A multi-professional team composed of clinicians and researchers from 14 centers distributed in the south, southeast, mid-west, northeast, and north regions of Brazil, who were knowledgeable in working with people with PD, built a multidimensional questionnaire to be used to conduct the interviews. The final version of the questionnaire, composed of 138 questions, was revised by a linguistic professional to adapt the language for people with PD from all socioeconomic levels. Most of the questions were self-reported daily life experience regarding a previous period (usual function over 2 weeks before social distancing had begun) and the current period (usual function over the past week, including the current day) of social distancing. The questionnaire includes scales and tests previously developed for PD, such as the Movement Disorder Society—Unified Parkinson's Disease Rating Scale (MDS-UPDRS), parts IB and II (12), Parkinson's Disease Questionnaire-8 (PDQ-8) (13), and probability of falling (14). The questionnaire was divided into two parts (Part 1 and Part 2—see the Supplementary Material), and each part lasted for approximately 30 min. Each part was applied on different days, and the interval between the interviews did not exceed 7 days.

To confirm the feasibility of the study, we applied the final version of the questionnaire in 40 people with PD (pilot study). Afterwards, the coordinator center trained the researchers involved in the study using videos (2 h), written material, and videoconferences to conduct the interviews with the participants. The participants were asked to indicate the best day and time for the interview by telephone and if a family member could help them to answer the questions.

On the first interview, the researchers applied Part 1 of the questionnaire that included 64 questions divided as follows: 1—general information, 2—socioeconomic status (six questions), 3—information associated with PD (i.e., perception of fear of falling, history of falls in the previous 12 months, self-reported freezing of gait and gait speed, and self-reported PD severity−12 questions), 4—information on access to medication and dosage (15) (six questions), 5—perception of health conditions related to COVID-19 (24 questions), and 6—perception of quality of life (PDQ-8) regarding the previous (eight questions) and the current (8 questions) period of social distancing.

On the second interview, the researchers applied Part 2 of the questionnaire that included 75 questions divided as follows: 1—self-reported physical activity volume (duration of session × frequency; see more details in Supplementary Material II), perception of physical activity volume, and routine previous and current period of social distancing (24 questions); 2—other adjuvant care for PD (four questions); 3—perception of emotional and mental health regarding the previous period of social distancing (seven questions); 4—perception of non-motor aspects of daily life experiences of PD (MDS-UPDRS Part IB—questions from 1.7 to 1.13) regarding the previous (seven questions) and the current (seven questions) period of social distancing; and 5—perception of motor aspects of daily life experiences of PD (MDS-UPDRS Part II) regarding the previous (13 questions) and the current (13 questions) period of social distancing.

We scored each question regarding previous perception of social distancing of the MDS-UPDRS-IB (seven questions), MDS-UPDRS-II (13 questions), PDQ-8 (eight questions), emotional and mental health (seven questions), and fear of falling (one question) from −2 to −1 [favorable perceptions of social distancing (FPSD)], 0 (neutral), and from 1 to 2 [unfavorable perceptions of social distancing (UPSD)], resulting in maximum negative and positive scores of −14 to +14, −26 to +26, −14 to +14, and −16 to +16, respectively. Negative scores indicate FPSD, and positive scores indicate UPSD.

### Statistical Analyses

Shapiro–Wilk and Levene's tests were used to assess data normality and homogeneity of variance, respectively.

We performed linear multiple regressions using the stepwise method to explain the variance of dependent variables (MDS-UPDRS-IB, MDS-UPDRS-II, and emotional and mental health). First, the univariate analyses were used to test which factors (self-reported changes in quality of life, physical activity volume, daily routine, PD severity, severity of freezing of gait, disease duration, medication dosage, fear of falling, probability of falling, fear of the COVID-19 pandemic, comorbidities, demographical characteristics, and socioeconomic status) would be associated with the dependent variables (MDS-UPDRS-IB, MDS-UPDRS-II, and emotional and mental health). Afterward, to explain the variance of the dependent variables, we included the factors in the linear multivariate analysis using the stepwise model if they presented a *P* ≤ 0.10 and a correlation of lower than 0.6 between them to avoid collinearity (16).

We used paired *t*-test to compare the physical activity volume (duration of session × frequency) between the previous and the current period of social distancing.

The results were presented as mean ± standard deviation. Statistical procedures were performed using the software SAS 9.2® (Institute Inc., Cary, NC, USA), and the level of significance was set at *P* ≤ 0.05.

## Results

### Participants

Only four participants dropped out of the study (two due to the low quality of the telephonic connection and two due to the discomfort with the answer about emotional status), and their information were not included in the final statistical analysis. The demographic characteristics, socioeconomic status, clinical perception, and perceptions related to social distancing and COVID-19 pandemic of the 478 people with PD are shown in Table 1.

**Table 1:** Demographic characteristics, socioeconomic status, clinical perception, and perceptions and implications of social distancing due to COVID-19 pandemic of the participants (mean, SD).

	**Participants (*n* = 478)**
**Demographic**	
Men/women (*n*)	255/167
Age (years)	67.27 (9.51)
Educational level (years)	11.12 (5.62)
**Socioeconomic status**	
A (high)	14.4%
B (middle)	40.3%
C (low)	37.4%
D–E (very low)	7.7%
**Clinical**	
Disease duration (years)	8.53 (6.27)
Estimated Hoehn and Yahr (H&Y) stage	2.21 (0.94)
H&Y 1	35.5%
H&Y 2	7.7%
H&Y 3	56.6%
**Self-reported freezing of gait**	
Yes	57.3%
No	42.6%
**Self-reported MDS-UPDRS-IB (scores)**	
UPSD	52.7%
Neutral (no difference)	35.5%
FPSD	11.7%
**Self-reported MDS-UPDRS-II (scores)**	
UPSD	60.0%
Neutral (no difference)	29.2%
FPSD	10.6%
**Self-reported emotional and mental health (scores)**	
UPSD	76.3%
Neutral (no difference)	15.9%
FPSD	7.7%
**Self-reported fear of falling (scores)**	
UPSD	46.6%
Neutral (no difference)	48.5%
FPSD	4.8%
**Self-reported PDQ-8 (score)**	
UPSD	49.7%
Neutral (no difference)	30.3%
FPSD	18.8%
_L_-Dopa-equivalent daily dose (mg/day)	629.32 (487.37)
**Change in medication dosage during social distancing**	
Yes	6.07%
No	93.3%
**Other diseases**	
None	27.6%
1	29.9%
2	23.65
3	11.0%
4 or more	7.7%
**Perceptions and implications of social distancing**	
Duration of social distancing (weeks)	7.70 (3.03)
**Probability of falling**	
Low	11.5%
Moderate	38.9%
High	49.5%
**Fear of the COVID-19 pandemic**	
I am not afraid	17.7%
I worry a little	35.7%
I worry a lot	46.4%
**Change in routine**	
No changes	13.8%
A little different	44.5%
Much different	41.6%
**Have you stopped other treatments for Parkinson's disease due to social distancing?**	
Yes	62.1%
No	37.8%
**If your response to the question above is “yes,” then what?**	
Voice therapy	60.3%
Psychologist	26.1%
Acupuncture	10.8%
Nursing guidance	1.1%
Occupational therapy	13.5%
Painting classes	5.9%
Other	35.3%
**Have you been practicing physical activities since the beginning of social distancing?**	
Yes	70.6%
No	29.7%
**If your response to the question above is “yes,” then how?**	
Self-guided	54.9%
Guided by a family member	10.7%
Distance-guided supervision from a professional	31.2%
Self-guided apps or TV shows	1.2%
Self-guided Internet (e.g., videos)	1.2%
**Perception of decreased physical activity volume**	
No changes	13.5%
Sometimes	0.9%
Almost always	14.4%
Always	62.7%

*FPSD, favorable perceptions of social distancing; UPSD, unfavorable perceptions of social distancing; MDS-UPDRS-IB, Movement Disorder Society—Unified Parkinson's Disease Rating Scale Part IB; MDS-UPDRS-II, Movement Disorder Society—Unified Parkinson's Disease Rating Scale Part II; PDQ-8, Parkinson's Disease Questionnaire-8; COVID-19, coronavirus disease 2019*.

Most of the participants reported middle (40%) and low (37%) socioeconomic status. In addition, most of the participants reported (a) moderate severity of PD (56%), (b) freezing of gait (57%), (c) no change in medication dosage (93%), (d) one disease (30%) other than PD (e.g., cardiovascular disease or diabetes), (e) high probability of falling (49%), and (f) changes in daily routine (44%). Although 70% of the participants have practiced physical activities (≥10 min in duration) since the beginning of social distancing, 63% had a high perception of decreased physical activity volume. However, 55% reported a self-guided physical activity, while only 31% reported a guided home-based physical activity with distance supervision by a health professional. No participant contracted COVID-19, but 46% reported fear of the COVID-19 pandemic (Table 1).

### Self-Reported Clinical Worsening Due to Social Distancing

Normality was checked by Shapiro–Wilk test, and the results suggested no apparent violation of the assumption (*p* > 0.100). Table 1 shows that most of the participants reported positive (UPSD) scores for the MDS-UPDRS-IB (52%), MDS-UPDRS-II (60%), emotional and mental health (76%), and PDQ-8 (76%). Although 48% of the participants reported no change (0 scores) for the fear of falling, 46% reported positive (UPSD) scores.

### Decreased Physical Activity Volume Due to Social Distancing

**Figure 1:**
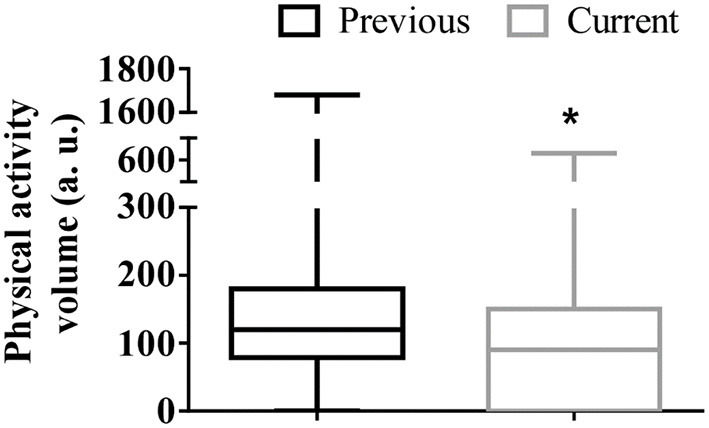
Box plots of the physical activity volume (duration of session × frequency) regarding the previous and current period of social distancing for the 478 people with Parkinson' disease. *Lower values in the current period of social distancing (*P* < 0.001). a.u., arbitrary unit.

Normality was checked by Shapiro–Wilk test, and the results suggested no apparent violation of the assumption (*p* > 0.200). There were significant differences (*P* < 0.001) between the previous (*M* = 174.68, *SD* = 202.17) and the current (*M* = 111.41, *SD* = 123.08) physical activity volume (duration of session × frequency) as shown in Figure 1.

### Factors of Influence on Self-Reported Clinical Worsening Due to Social Distancing

Table 2 shows the variables that explained the self-reported changes in the MDS-UPDRS-IB scores, MDS-UPDRS-II scores, and emotional and mental health scores according to linear multiple regressions (stepwise method).

**Table 2:** Linear multiple regressions (stepwise method) with included factors and MDS-UPDRS-IB, MDS-UPDRS-II, and emotional and mental health scores as dependent variables.

			**Variance explained (adjusted model** ***R*** ^**2**^ **change)**	
**Independent factors**	**Partial** ***R*** ^2^	**Model** ***R*** ^2^ **Change**	***F value***	***P value***
**MDS-UPDRS-IB**				
Whole model			0.23	
SRC in PDQ-8 (scores)	0.215	0.22	131.95	<0.001
SRC in daily routine (scores)	0.012	0.23	70.68	<0.007
SCR in fear of falling (scores)	0.011	0.24	50.23	<0.007
**MDS-UPDRS-II**				
Whole model			0.22	0.69
SRC in PDQ-8 (scores)	0.211	0.21	128.90	<0.001
SRC in FOG (scores)	0.017	0.23	71.21	<0.001
SCR in fear of falling (scores)	0.009	0.23	50.01	0.013
**Emotional and mental health**				
Whole model			0.18	
SRC in PDQ-8 (scores)	0.172	0.17	99.86	<0.001
SRC in PA volume (a.u.)	0.014	0.18	54.82	<0.005
SCR in probability of falling (scores)	0.009	0.19	38.82	0.017

*SRC, self-reported change; PDQ-8, Parkinson's Disease Questionnaire-8; FOG, freezing of gait; PA, physical activity; a.u., arbitrary unit*.

The self-reported changes in quality of life, daily routine, and fear of falling explained 23% (*P* < 0.05) of the variance in the MDS-UPDRS-IB scores.

The self-reported changes in quality of life, freezing of gait severity, and fear of falling explained 22% (*P* < 0.05) of the variance in the MDS-UPDRS-II scores.

Finally, the self-reported changes in quality of life, physical activity volume, and probability of falling explained 18% (*P* < 0.05) of the variance in the emotional and mental health scores.

## Discussion

To the best of our knowledge, this is the first study to show that (a) people with PD living in Brazil during social distancing due to COVID-19 pandemic showed a self-reported clinical worsening (MDS-UPDRS-IB, MDS-UPDRS-II, and emotional and mental health scores), and a self-reported decline in the quality of life and in the physical activity volume; (b) the self-reported decline in the quality of life was a common predictor of self-reported clinical worsening (MDS-UPDRS-IB, MDS-UPDRS-II, and emotional and mental health scores); (c) the self-reported increase in fear of falling was the common predictor of MDS-UPDRS-IB and MDS-UPDRS-II scores, but the self-reported changes in daily routine and freezing of gait severity also entered the regression model to explain the variance in the MDS-UPDRS-IB and MDS-UPDRS-II scores, respectively; and (d) only the self-reported decline in the physical activity volume explained the variance in the emotional and mental health scores.

Even though a lockdown has not been implemented in Brazil, our results are consistent with the previous findings for people with PD living in India, where a lockdown had been in place for barely 3 weeks (4), therefore shorter than the 7 weeks of social distancing experienced by the participants in the present study. People with PD living in Italy likewise reported an acute clinical worsening in either motor disturbances or neuropsychiatric non-motor symptoms due to lockdown and infection outbreak (2), consistent with clinical worsening in mental health and a reduced physical activity level in people with PD living in Egypt (3) and in Germany (5). However, according to the present results, the detrimental effects of social distancing on the motor and non-motor aspects of PD were more severe in Brazil. While only 10% of people with PD living in India (4), 28% in Italy (2), and 31% in Germany (2) reported some clinical worsening, more than 50% of the participants in the present study reported a deterioration in the motor and non-motor aspects of the daily life experience of PD (Table 1). Such result is alarming, although it can be explained in part due to the larger number of participants in the present study.

Although a self-reported decline in the quality of life was a common predictor of self-reported clinical worsening (MDS-UPDRS-IB, MDS-UPDRS-II, and emotional and mental health scores), the self-reported changes in fear of falling, daily routine, freezing of gait severity, probability of falling, and physical activity volume were also predictors of self-reported clinical worsening. These findings are in accordance with previous evidence (17–21), but we demonstrate, for the first time, the complex relationship between the multidimensional aspects caused by PD during social distancing.

Interestingly, only the self-reported decline in the physical activity volume was a predictor of self-reported decline in the emotional and mental health scores. The decreased physical activity level during social distancing has been appointed as one of the main causes of poor mental health (8, 22–24), followed by pandemic fear (25), and sedentary behavior (26). In fact, the best available evidence recommends increasing physical activity levels in this population to alleviate PD severity and to improve mental health (27). Although most of the participants (55%) have reported practicing self-guided physical activities at home since the beginning of social distancing, its volume was lower than the previous social distancing (Figure 1). In addition, recent evidence has shown that a long-term, high-intensity exercise program (over 2 years) may enhance the maintenance of health over time in people with PD (28) even when the training is home-based and remotely supervised (29). In this sense, it is possible to suggest that not only home-based physical activities supervised for people with PD during the pandemic are necessary but also a long-term, high-volume exercise program to avoid poor mental health during social distancing.

Furthermore, findings from a meta-analysis suggest that supervised training at facilities produces more long-term gains in gait and balance than home-based training in people with PD (29). Among the participants who were practicing some physical activity at home, only 31% of the participants reported a guided home-based physical activity with distance supervision. Taken together, the lack of proper supervision and self-reported decrease in the volume of physical activity could explain the self-reported clinical worsening in the present study, although 55% of the participants reported a self-guided physical activity.

It is important to highlight that the self-reported changes in daily routine during social distancing were able to predict the self-reported changes of non-motor aspects (e.g., sleep problems, pain, fatigue, and daytime sleepiness) of the daily life experiences (MDS-UPDRS-IB scores) of people with PD. Thus, our results demonstrate, for the first time, a direct association between daily routine impoverishment and self-reported changes in non-motor aspects due to social distancing. The interruption in multi-professional care is a critical part of the changes in daily routine, as most of the participants reported interruption in one or more adjuvant treatments for PD, for example, physiotherapy and occupational therapy (Table 1). On the other hand, a smaller number of the participants reported some cancellation in medical care, probably because the interval between medical care appointments is longer than in allied healthcare appointments (Table 1). The lack of multi-professional care can directly and negatively affect the non-motor symptoms of PD and, consequently, the quality of life due to increased stress associated with the perception of a disruption of the supportive care network, which is considered fundamental in PD treatment (30).

Currently, the eminent challenge is to provide adequate ongoing care for people with PD to face the necessary self-isolation and social distancing (31). In fact, in high-income countries, there has been a widespread and rapid adoption of telemedicine and healthcare technology for people with PD and adequate resources (8, 10, 31). Despite the limited number of evidence, telemedicine has been considered able to achieve comparable outcomes than regular in-person visits (10, 32). Thus, further studies investigating the impact of social distancing and its consequences on the motor and non-motor aspects in people with PD are urgently needed. That is particularly required in low-income countries (e.g., Brazil), where telemedicine and e-health systems are not largely available (32) and the socioeconomic differences among the regions are huge. Telemedicine requires, besides additional technological implementation, professionals with proper training, among the other challenges (10). Our findings suggest that people with PD require not only telemedicine care but also “e-health systems and multi-professional care” in facing the need for continued restrictions on social life for months or even years to come until a vaccine is found. Thus, we believe that the results of this study offer a realistic and broad landscape on the impact of social distancing in people with PD living in Brazil. This landscape may guide better public policy to support this frail population.

The present study has positive aspects and some limitations. The benefits of multicenter data include a larger number of participants, from different geographic locations, and the inclusion of people with PD at different stages, increasing the external validity of the study. In addition, we included at least two cities from each Brazilian region, as Brazil is a continental country with substantial socioeconomic differences. Other positive aspects of this study include (a) only the inclusion of people with PD who are users of the health services from all centers, in order to ensure the PD diagnosis, (b) the use of a multidimensional questionnaire based on gold-standard tests developed for PD, and (c) the interview by phone in order to reduce the interference of the educational level. The limitations of this study include (a) its cross-sectional nature, preventing the determination of a cause-and-effect relationship—further longitudinal studies are needed to confirm the suggested relationships; (b) the absence of a control group (age- and sex-matched healthy controls), which would allow elucidating if the PD population is more vulnerable to social distancing or not—this question should be answered in further studies; (c) a proper cognitive screening, for example, using the Montreal Cognitive Assessment—however, we considered the ability of the participants to properly answer questions about personal and socio-economic information and confirmed their answers with the answers of the caregiver; and (d) we used MDS-UPDRS, which was not validated at that time for telephone interviews. However, according to MDS recommendation, sections IB and II have been designed to be amenable to a patient/caregiver questionnaire format and therefore can be completed without the presence of the investigator. Based on this recommendation, we assume that we could include these two sections in the present study.

In conclusion, most of the people with PD reported a clinical worsening in non-motor and motor aspects of daily life experiences and emotional and mental health due to social distancing. Self-reported changes in quality of life, freezing of gait, decreased physical activity volume, daily routine, and fear of falling explained the self-reported clinical worsening. The negative impact of social distancing on the multidimensional aspects of PD in people living in Brazil compared with those in other countries may have been aggravated by the precarious e/tele-health system.

## Data Availability Statement

The raw data supporting the conclusions of this article will be made available by the authors, without undue reservation.

## Ethics Statement

The studies involving human participants were reviewed and approved by University of São Paulo. The patients/participants provided their written informed consent to participate in this study.

## Author Contributions

MP: study supervision. CS-B, DC, RCFJ, LA, AG, KN, HS, AL, VI, HK, RG, NB, RB, CC, MF, FS, and MP: study concept and design, acquisition of data, critical revision of the manuscript for important intellectual content, and administrative, technical, or material support. MP, CS-B, and DC: analysis and interpretation of data. CS-B: writing of the first draft. DC and MP: drafting of the manuscript. DC and CS-B: statistical analysis. All authors contributed to the article and approved the submitted version.

## Conflict of Interest

The authors declare that the research was conducted in the absence of any commercial or financial relationships that could be construed as a potential conflict of interest.

## Publisher's Note

All claims expressed in this article are solely those of the authors and do not necessarily represent those of their affiliated organizations, or those of the publisher, the editors and the reviewers. Any product that may be evaluated in this article, or claim that may be made by its manufacturer, is not guaranteed or endorsed by the publisher.
