# Supplementary material for: Multidimensional Factors Can Explain the Clinical Worsening in People With Parkinson's Disease During the COVID-19 Pandemic: A Multicenter Cross-Sectional Trial
Source: Front Neurol. 2021 Jul 30;12:708433. doi: 10.3389/fneur.2021.708433 (PMC8362931; doi:10.3389/fneur.2021.708433)
Supplement: Supplementary file 1 [file Data_Sheet_1.DOCX]

**QUESTIONNAIRE FOR INVESTIGATING THE IMPACT OF SOCIAL DISTANCING ON**

**PARKINSON’S DISEASE**

***PART I***

Interviewer:

Date:

Reported by:

( ) patient ( ) family ( ) caregiver

**PERSONAL INFORMATION**

Date: Name:

ID: Gender: ( ) Male ( ) Female Date of birth Age:

**SOCIOECONOMIC CONDITIONS**

1 - How high is the educational level of the head of the family? Consider as the head of the household the person who most contributes to the family income.

(1) Illiterate / pre-kindergarten

(2) Complete elementary school / Secondary education incomplete

(3) Complete Elementary / High school (middle / junior) incomplete

(4) Complete high school / incomplete college

(5) University Degree

2 - Number of years you attended school: ( )

3 – Considering the street where you live, you would say the street is:

(1) Asphalted streets / Paved street

(2) Dirt street / Gravel street

4 - Where does the water used in your current home come from?

(1) Water general distribution

(2) Water well/headwater

(3) Any other method

5 - COMFORT ITEMS YOU HAVE AT HOME

Now I am going to ask you some questions about things and resources in your current home. All the things I am going to talk to you should be working, including those that are stored [stored and unused product]. If they are not working, speak only about those you intend to repair or replace within the next six months.

5.1. Number of passenger cars for private use only –

5.2. Number of monthly employees [who receive a monthly salary], considering only those who work, at least, five days a week –

5.3. Number of washing machines, excluding [not counting] Portuguese hand washing tank –

5.4. Number of bathrooms –

5.5. Number of devices that play CD or DVD, including any device [equipment] playing DVD, does not consider the car CD or DVD player -

5.6. Number of refrigerators –

5.7. Number of independent freezers or part of the duplex refrigerator –

5.8. Number of microcomputers, considering desktop computers, laptops, notebooks and netbooks, and disregarding tablets, iPad, or smartphones –

5.9. Number of dishwashers –

5.10. Number of microwave ovens –

5.11. Number of motorcycles (do not count those that are used exclusively for professional use or job) –

5.12. Number of clothes drying machines, including washer and dryer types –

6. Concerning your family income, after the beginning of the COVID-19 pandemic, it is:

(0) Same

(1) Slightly lower

(2) Much lower

(3) Slightly higher

(4) Much higher

**INFORMATION RELATED TO PARKINSON’S DISEASE**

7. How long ago did you receive the clinical diagnosis of Parkinson's disease?

8. Regarding your work position:

(1) I am still working

(2) I am retired

(3) I am working in domestic services

(4) I am unemployed

9. Do you attend any association for patients?

( ) Yes

( ) No

10. Currently, who helps you in your care?

(1) Nobody, I take care of myself

(2) My wife / husband

(3) My son / daughter

(4) I pay someone to do it

(5) Another person

11. BEFORE the beginning of the social distancing, who helped in your care?

(1) Nobody, I take care of myself

(2) My wife / husband

(3) My son / daughter

(4) I pay someone todo it

(5) Another person

12. In which side of your body the first symptoms of Parkinson's Disease started?

( ) Right

( ) Left

13. Currently, do your symptoms related to Parkinson’s disease remain on one side or they affect both sides of your body?

(1) Symptoms remain only on one side

(2) Symptoms are present on both sides

14. Did you realize that with the disease progression [as the disease progressed] your balance also started to get worse?

( ) Yes

( ) No

15. Have you fallen in the past 12 months?

( ) Yes

( ) No

16. Did you experience freezing of gait while walking (feet get glued to the floor) in the past month?

( ) Yes

( ) No

17. Comparing yourself to age-matched people (your wife or brother for example), but thinking of people who do NOT [stress] have Parkinson's or other health conditions like stroke, would you say that your usual walking speed is:

(1) Faster than a person without Parkinson’s

(0) The same of a person without Parkinson’s

(-1) Slower than a person without Parkinson’s

18. Have you been worried or afraid of falling? If so, how is that feeling?

(1) Slightly more worried than before the beginning of social distancing

(2) Much more worried than before the beginning of social distancing

(0) The same concern before the beginning of social distancing

(-1) Slightly less worried than before the beginning of social distancing

(-2) Much less worried than before the beginning of social distancing

**INFORMATION CONCERNING ACCESS TO THE MEDICINE DRUGS**

19. What medications, and in which dosage, do you take for the treatment of Parkinson's disease? Since when do you take these drugs? Has there been any change since the beginning of the isolation?

20. Do you take any medication other than the ones to Parkinson's?

( ) Yes

( ) No

21. Have you been able to buy / get the medicines to treat Parkinson's disease after the beginning of social distancing?

( ) Yes

( ) No

22. Who buys / takes your medicines?

(1) Myself

(2) My wife / husband

(3) My son / daughter

(4) Others

23. Did you find any new difficulties to buy / get the medication?

( ) Yes

( ) No

24. If so, what?

(1) It had to be delivered at home

(2) I asked someone to get it for me

(3) I didn't have money to buy it

(4) It is harder to get a doctor's prescription

(5) The queue was very long at the place where I get / buy the medicine

(6) The pharmacy / place I usually withdraw my medicine was closed

(7) The medication was unavailable at the pharmacy where I withdraw my medicine

**GENERAL HEALTH – INFORMATION CONCERNING COVID-19**

25. Do you think you are well informed about the problems caused by the new Coronavirus / COVID -19?

( ) Yes

( ) No

26. Where do you usually get the news related to the COVID-19?

(1) Television

(2) Radio

(3) Internet

(4) WhatsApp / Facebook

(5) Printed newspaper

(6) I don't see anything, I just talk to my accompanying person / caregiver

27. Have you had COVID-19 PROVEN WITH THE TEST?

( ) Yes

( ) No

28. Are you with COVID-19 PROVEN WITH TEST?

( ) Yes

( ) No

29. Does anyone in your family have or had COVID-19 TESTED?

( ) Yes

( ) No

30. If so, whom?

31. Are you afraid to get COVID-19?

(0) I am afraid

(1) I worry a little

(2) I worry a lot

32. What is your main fear?

(1) To die

(2) To be admitted to a hospital

(3) Breathing by artificial respiratory apparatus

(4) Have to be isolated from family

33. How many weeks have you been isolated in quarantine?

34. Have you gone out of your home?

( ) Yes

( ) No

35. How often have you gone out of your home?

(1) Everyday

(2) 2-3 times / week

(3) Once a week

(4) Once every 15 days

(5) Once a month

36. When you leave home to go somewhere (market, pharmacy, etc.), you use:

(1) Own car or from a family member

(2) Taxi / Uber / App

(3) Bus / Subway

(4) Walk

(5) Cycle

37. What is the purpose when you go out to go somewhere?

(1) To buy medicine

(2) To buy food

(3) To Walk

(4) To go to physical therapy / speech therapy / physician

(5) To walking

(6) To Stroll through the streets and squares

(7) Any other purpose

38. What activities do you miss the most about going out of your home?

(1) To get out for some shopping

(2) To get out for therapies

(3) To get out to visit Church

(4) To get out to visit family members

(5) To get out to stroll through / walking

39. When you leave home to go anywhere, do you wear a mask?

( ) Yes

( ) No

40. If not, why?

(1) I do not have one

(2) I do not feel good

(3) I do not breathe well

(4) People do not understand when I am speaking

(5) I am ashamed

41. Do you wash your hands when you get back home?

( ) Yes

( ) No

42. Do you have heart disease?

( ) Yes

( ) No

43. Do you have blood hypertension (high blood pressure)?

( ) Yes

( ) No

44. Do you have diabetes (blood sugar problem)?

( ) Yes

( ) No

45. Do you have dyslipidemia (cholesterol problem)?

( ) Yes

( ) No

46. Do you have respiratory disease, any breathing problems (asthma, bronchitis or something else)?

( ) Yes

( ) No

47. Do you have any other disease that I did not ask you yet?

( ) Yes

( ) No

If so, what?

**QUALITY OF LIFE**

Mobility

48-Have you had difficulty getting around in public?

(0) Never

1. Occasionally
2. Sometimes
3. Often
4. Always

49 - In comparison with the two weeks BEFORE social distancing, this difficulty getting around in public:

(1) Is a little worse

(2) Is much worse

(0) Hasn’t changed

(-1) Is a little better

(-2) Is much better

Activities of Daily Living

50- Have you had difficulty dressing yourself?

(0) Never

1. Occasionally
2. Sometimes
3. Often
4. Always

51- In comparison with the two weeks BEFORE social distancing, the difficulty dressing yourself:

(1) Is a little worse

(2) Is much worse

(0) Hasn’t changed

(-1) Is a little better

(-2) Is much better

Emotional well being

52- Have you felt depressed:

(0) Never

1. Occasionally
2. Sometimes
3. Often
4. Always

53- In comparison with the two weeks BEFORE social distancing, depression:

(1) Is a little worse

(2) Is much worse

(0) Hasn’t changed

(-1) Is a little better

(-2) Is much better

Estigma/Prejudice

54- Have you felt embarrassed in public?

(0) Never

1. Occasionally
2. Sometimes
3. Often
4. Always

55- In comparison with the two weeks BEFORE social distancing, this embarrassment:

(1) Is a little worse

(2) Is much worse

(0) Hasn’t changed

(-1) Is a little better

(-2) Is much better

Social support/relationships

56- Have you had problems with your close personal relationship?

(0) Never

1. Occasionally
2. Sometimes
3. Often
4. Always

57- In comparison with the two weeks BEFORE social distancing, the relationship with people close to you:

(1) Is a little worse

(2) Is much worse

(0) Hasn’t changed

(-1) Is a little better

(-2) Is much better

Cognition - comprehension/ thoughts

58- Have you had with your concentration (for example, reading or watching tv)?

(0) Never

1. Occasionally
2. Sometimes
3. Often
4. Always

59- In comparison with the two weeks BEFORE social distancing, concentration problems:

(1) Is a little worse

(2) Is much worse

(0) Hasn’t changed

(-1) Is a little better

(-2) Is much better

Communication

60- Have you ever felt you couldn’t communicate well (people couldn't hear or understand you)?

(0) Never

1. Occasionally
2. Sometimes
3. Often
4. Always

61- In comparison with the two weeks BEFORE social distancing, communication problems:

(1) Is a little worse

(2) Is much worse

(0) Hasn’t changed

(-1) Is a little better

(-2) Is much better

Bodily discomfort

62 - Have you had painful muscle cramps or spasms (involuntary muscle movements)?

(0) Never

1. Occasionally
2. Sometimes
3. Often
4. Always

63- In comparison with the two weeks BEFORE social distancing, these cramps or involuntary movements:

(1) Is a little worse

(2) Is much worse

(0) Hasn’t changed

(-1) Is a little better

(-2) Is much better

***PART II***

Physical Activity Level

64- Do you practice any kind of physical activity (physiotherapy, dance, others)?
( ) yes

( ) no

65- For how long have you been practicing physical activities (in months)?

66- Which modalities?

67- Did you pay for any of them?

( ) yes

( ) no

68- What were the duration of your practice (in minutes)?

69- How many times a week?

70- Regarding the intensity of the activities, do you consider they were:
(1) Mild: There is no need for an effort, and you can breathe normally

(2) Moderate: Some effort is needed and you have to breathe a little bit harder than you normally do

(3) Intense: A big effort is needed and you have to breathe a lot harder than you normally do

71- Did you have to stop any other activity you used to do to help in your Parkinson’s disease treatment?

( ) yes

( ) no

72- If yes which one?

73- Why did you have to stop?

1. It was canceled
2. I was scared to continue
3. I can’t go

74- Have you been practicing physical actives since the begging of social distancing?

( ) yes

( ) no

75- If yes, for how long?

1. Since the beginning of social distancing
2. 1-2 weeks
3. A month

76- If not, why?

1. I don’t know what I could do
2. I don’t feel like doing it
3. There is no one who could help me
4. I don’t have time
5. I don’t have enough space
6. I’m scared

77- Which modalities have you been practicing?

78- For how long (in minutes)?

79 - How many times a week?

80 - How?

1. By myself
2. With guidance from a family member
3. With distance guidance from a professional
4. Using apps or tv shows to help me
5. Using videos from the internet

81- If you have help from someone or use an app or video, do you pay for it?

( ) yes

( ) no

82 Regarding your physical activities, do you think they are the same as they were before social distancing?

1. no
2. Sometimes
3. Almost always
4. Always

83- Regarding the intensity of the activities you do now, at home, do you consider they are:
(1) Mild: There is no need for an effort, and you can breathe normally

(2) Moderate: Some effort is needed and you have to breathe a little bit harder than you normally do

(3) Intense: A big effort is needed and you have to breathe a lot harder than you normally do

84- Where do you practice?

1. Living room
2. Bedroom
3. backyard
4. Balcony
5. garage
6. outside

85- In comparison with your daily routine two weeks before the beginning of social distancing, your routine now is:

(0) the same

1. a little different
2. much different

86- What were the main changes?

87- Among those changes, which one was the hardest for you?

**OTHER CARE FOR PARKINSON’S DISEASE**

88- Did you have to stop any other treatment, that you used to do before the beginning of social distancing, for your Parkinson's disease treatment?

( ) yes

( ) no

89- If yes, which one?

1. voice therapy
2. psychologist
3. Acupuncture
4. Nursing guidance
5. Occupational therapy
6. painting classes
7. other - which?

90- Your Parkinson’s disease medical doctor is?

1. private care
2. health insurance
3. public care

91- What about medical appointments? Since the beginning of social distancing have you had any medical consultation?

1. yes
2. no, I canceled it
3. no, I was too scared to go
4. no, it was canceled by the doctor/hospital
5. I didn’t have any medical appointment scheduled

**EMOTIONAL AND MENTAL HEALTH**

In comparison with how you were felling two weeks BEFORE the beginning of social distancing, nowadays you:

92- Have you been felling lack of interest to realize actives at home or to talk to people:

(1) Is a little worse

(2) Is much worse

(0) Hasn’t changed

(-1) Is a little better

(-2) Is much better

93- Have you been feeling nervous, tense, preoccupied, or anxious:

(1) Is a little worse

(2) Is much worse

(0) Hasn’t changed

(-1) Is a little better

(-2) Is much better

94- Have you been feeling discouragement, sadness, despair, or having lonely feelings:

(1) Is a little worse

(2) Is much worse

(0) Hasn’t changed

(-1) Is a little better

(-2) Is much better

95- Have you been feeling that it is hard to adapt to changes in your life:

(1) Is a little worse

(2) Is much worse

(0) Hasn’t changed

(-1) Is a little better

(-2) Is much better

96- How do you fell about your memory:

(1) Is a little worse

(2) Is much worse

(0) Hasn’t changed

(-1) Is a little better

(-2) Is much better

97- How do you feel about your attention:

(1) Is a little worse

(2) Is much worse

(0) Hasn’t changed

(-1) Is a little better

(-2) Is much better

98- How do you feel about your reasoning:

(1) Is a little worse

(2) Is much worse

(0) Hasn’t changed

(-1) Is a little better

(-2) Is much better

**MDS-UPDRS – PART IB**

99. SLEEP PROBLEMS

Over the past week, have you had trouble going to sleep at night or staying asleep

through the night? Consider how rested you felt after waking up in the morning.

0: Normal: No problems.

1: Slight: Sleep problems are present but usually do not cause trouble

getting a full night of sleep.

2: Mild: Sleep problems usually cause some difficulties getting a full night

of sleep.

3: Moderate: Sleep problems cause a lot of difficulties getting a full night of

sleep, but I still usually sleep for more than half the night.

4: Severe: I usually do not sleep for most of the night.

100. In comparison with the two weeks BEFORE social distancing, in the last week, these sleep problems

(1) Is a little worse

(2) Is much worse

(0) Hasn’t changed

(-1) Is a little better

(-2) Is much better

101. DAYTIME SLEEPINESS

Over the past week, have you had trouble staying awake during the daytime?

0: Normal: No daytime sleepiness.

1: Slight: Daytime sleepiness occurs but I can resist and I stay awake.

2: Mild: Sometimes I fall asleep when alone and relaxing. For example,

while reading or watching TV.

3: Moderate: I sometimes fall asleep when I should not. For example, while

eating or talking with other people.

4: Severe: I often fall asleep when I should not. For example, while eating or

talking with other people.

102. In comparison with the two weeks BEFORE social distancing, in the last week, the daytime sleeping (feel sleepy during the day)

(1) Is a little worse

(2) Is much worse

(0) Hasn’t changed

(-1) Is a little better

(-2) Is much better

103. PAIN AND OTHER SENSATIONS

Over the past week, have you had uncomfortable feelings in your body like pain, aches tingling or cramps?

0: Normal: No uncomfortable feelings.

1: Slight: I have these feelings. However, I can do things and be with other

people without difficulty.

2: Mild: These feelings cause some problems when I do things or am with

other people.

3: Moderate: These feelings cause a lot of problems, but they do not stop me

from doing things or being with other people.

4: Severe: These feelings stop me from doing things or being with other

people.

104. In comparison with the two weeks BEFORE social distancing, in the last week, these uncomfortable feelings

(1) Is a little worse

(2) Is much worse

(0) Hasn’t changed

(-1) Is a little better

(-2) Is much better

105. URINARY PROBLEMS

Over the past week, have you had trouble with urine control? For example, an urgent need to urinate, a need to urinate too often, or urine accidents?

0: Normal: No urine control problems.

1: Slight: I need to urinate often or urgently. However, these problems do

not cause difficulties with my daily activities.

2: Mild: Urine problems cause some difficulties with my daily activities.

However, I do not have urine accidents.

3: Moderate: Urine problems cause a lot of difficulties with my daily activities,

including urine accidents.

4: Severe: I cannot control my urine and use a protective garment or have a

bladder tube.

106. In comparison with the two weeks BEFORE social distancing, in the last week, currently, these urinary problems

(1) Is a little worse

(2) Is much worse

(0) Hasn’t changed

(-1) Is a little better

(-2) Is much better

107. CONSTIPATION PROBLEMS

Over the past week have you had constipation troubles that cause you difficulty

moving your bowels?

0: Normal: No constipation.

1: Slight: I have been constipated. I use extra effort to move my bowels.

However, this problem does not disturb my activities or my being

comfortable.

2: Mild: Constipation causes me to have some troubles doing things or

being comfortable.

3: Moderate: Constipation causes me to have a lot of trouble doing things or

being comfortable. However, it does not stop me from doing

anything.

4: Severe: I usually need physical help from someone else to empty my

bowels.

108. In comparison with the two weeks BEFORE social distancing, in the last week, these constipation problems

(1) Is a little worse

(2) Is much worse

(0) Hasn’t changed

(-1) Is a little better

(-2) Is much better

109. LIGHT HEADEDNESS ON STANDING

Over the past week, have you felt faint, dizzy or foggy when you stand up after sitting or lying down?

0: Normal: No dizzy or foggy feelings.

1: Slight: Dizzy or foggy feelings occur. However, they do not cause me

troubles doing things.

2: Mild: Dizzy or foggy feelings cause me to hold on to something, but I do

not need to sit or lie back down.

3: Moderate: Dizzy or foggy feelings cause me to sit or lie down to avoid

fainting or falling.

4: Severe: Dizzy or foggy feelings cause me to fall or faint.

110. In comparison with the two weeks BEFORE social distancing, in the last week, these dizzy feelings

(1) Is a little worse

(2) Is much worse

(0) Hasn’t changed

(-1) Is a little better

(-2) Is much better

111. FATIGUE

Over the past week, have you usually felt fatigued? This feeling is not part of being

sleepy or sad.

0: Normal: No fatigue.

1: Slight: Fatigue occurs. However it does not cause me troubles doing

things or being with people.

2: Mild: Fatigue causes me some troubles doing things or being with

people.

3: Moderate: Fatigue causes me a lot of troubles doing things or being with

people. However, it does not stop me from doing anything.

4: Severe: Fatigue stops me from doing things or being with people.

112. In comparison with the two weeks BEFORE social distancing, in the last week, this feeling of tiredness or fatigue

(1) Is a little worse

(2) Is much worse

(0) Hasn’t changed

(-1) Is a little better

(-2) Is much better

**MDS-UPDRS – PART II**

113. SPEECH

Over the past week, have you had problems with your speech?

0: Normal: Not at all (no problems).

1: Slight: My speech is soft, slurred or uneven, but it does not cause others

to ask me to repeat myself.

2: Mild: My speech causes people to ask me to occasionally repeat

myself, but not everyday.

3: Moderate: My speech is unclear enough that others ask me to repeat myself

every day even though most of my speech is understood.

4: Severe: Most or all of my speech cannot be understood.

114. In comparison with the two weeks BEFORE social distancing, in the last week, these problems with your speech

(1) Is a little worse

(2) Is much worse

(0) Hasn’t changed

(-1) Is a little better

(-2) Is much better

115. SALIVA AND DROOLING

Over the past week, have you usually had too much saliva during when you are

awake or when you sleep?

0: Normal: Not at all (no problems).

1: Slight: I have too much saliva, but do not drool.

2: Mild: I have some drooling during sleep, but none when I am awake.

3: Moderate: I have some drooling when I am awake, but I usually do not need

tissues or a handkerchief.

4: Severe: I have so much drooling that I regularly need to use tissues or a

handkerchief to protect my clothes.

116. In comparison with the two weeks BEFORE social distancing, in the last week, these problems

(1) Is a little worse

(2) Is much worse

(0) Hasn’t changed

(-1) Is a little better

(-2) Is much better

117. CHEWING AND SWALLOWING

Over the past week, have you usually had problems swallowing pills or eating meals?

Do you need your pills cut or crushed or your meals to be made soft, chopped or

blended to avoid choking?

0: Normal: No problems.

1: Slight: I am aware of slowness in my chewing or increased effort at

swallowing, but I do not choke or need to have my food specially

prepared.

2: Mild: I need to have my pills cut or my food specially prepared because

of chewing or swallowing problems, but I have not choked over

the past week.

3: Moderate. I choked at least once in the past week.

4: Severe: Because of chewing and swallowing problems, I need a feeding

tube.

118. In comparison with the two weeks BEFORE social distancing, in the last week, these chewing and swallowing problems

(1) Is a little worse

(2) Is much worse

(0) Hasn’t changed

(-1) Is a little better

(-2) Is much better

119. EATING TASKS

Over the past week, have you usually had troubles handling your food and using

eating utensils? For example, do you have trouble handling finger foods or using

forks, knives, spoons, chopsticks?

0: Normal: Not at all (no problems).

1: Slight: I am slow, but I do not need any help handling my food and have

not had food spills while eating.

2: Mild: I am slow with my eating and have occasional food spills. I may

need help with a few tasks such as cutting meat.

3: Moderate: I need help with many eating tasks but can manage some alone.

4: Severe: I need help for most or all eating tasks.

120. In comparison with the two weeks BEFORE social distancing, in the last week, currently, these troubles handling your food

(1) Is a little worse

(2) Is much worse

(0) Hasn’t changed

(-1) Is a little better

(-2) Is much better

121. DRESSING

Over the past week, have you usually had problems dressing? For example, are you slow or do you need help with buttoning, using zippers, putting on or taking off your clothes or jewelry?

0: Normal: Not at all (no problems).

1: Slight: I am slow but I do not need help.

2: Mild: I am slow and need help for a few dressing tasks (buttons,

bracelets).

3: Moderate: I need help for many dressing tasks.

4: Severe: I need help for most or all dressing tasks.

122. In comparison with the two weeks BEFORE social distancing, in the last week, these problems dressing

(1) Is a little worse

(2) Is much worse

(0) Hasn’t changed

(-1) Is a little better

(-2) Is much better

123. HYGIENE

Over the past week, have you usually been slow or do you need help with washing,

bathing, shaving, brushing teeth, combing your hair or with other personal hygiene?

0: Normal: Not at all (no problems).

1: Slight: I am slow but I do not need any help.

2: Mild: I need someone else to help me with some hygiene tasks.

3: Moderate: I need help for many hygiene tasks.

4: Severe: I need help for most or all of my hygiene tasks.

124. In comparison with the two weeks BEFORE social distancing, in the last week, these problems washing, bathing, shaving, brushing teeth

(1) Is a little worse

(2) Is much worse

(0) Hasn’t changed

(-1) Is a little better

(-2) Is much better

125. HANDWRITING

Over the past week, have people usually had trouble reading your handwriting?

0: Normal: Not at all (no problems).

1: Slight: My writing is slow, clumsy or uneven, but all words are clear. Some words are unclear and difficult to read.

2: Mild: Some words are unclear and difficult to read.

3: Moderate: Many words are unclear and difficult to read.

4: Severe: Most or all words cannot be read.

126. In comparison with the two weeks BEFORE social distancing, in the last week, these handwriting problems:

(1) Is a little worse

(2) Is much worse

(0) Hasn’t changed

(-1) Is a little better

(-2) Is much better

127. DOING HOBBIES AND OTHER ACTIVITIES

Over the past week, have you usually had trouble doing your hobbies or other things that you like to do?

0: Normal: Not at all (no problems).

1: Slight: I am a bit slow but do these activities easily.

2: Mild: I have some difficulty doing these activities.

3: Moderate: I have major problems doing these activities, but still do most.

4: Severe: I am unable to do most or all of these activities.

128. In comparison with the two weeks BEFORE social distancing, in the last week, this trouble doing your hobbies or other things that you like to do:

(1) Is a little worse

(2) Is much worse

(0) Hasn’t changed

(-1) Is a little better

(-2) Is much better

129. TURNING IN BED

Over the past week, do you usually have trouble turning over in bed?

0: Normal: Not at all (no problems).

1: Slight: I have a bit of trouble turning, but I do not need any help.

2: Mild: I have a lot of trouble turning and need occasional help from someone else.

3: Moderate: To turn over I often need help from someone else.

4: Severe: I am unable to turn over without help from someone else.

130. In comparison with the two weeks BEFORE social distancing, in the last week, this trouble turning in bed:

(1) Is a little worse

(2) Is much worse

(0) Hasn’t changed

(-1) Is a little better

(-2) Is much better

131. TREMOR

Over the past week, have you usually had shaking or tremor?

0: Normal: Not at all. I have no shaking or tremor.

1: Slight: Shaking or tremor occurs but does not cause problems with any activities.

2: Mild: Shaking or tremor causes problems with only a few activities.

3: Moderate: Shaking or tremor causes problems with many of my daily activities.

4: Severe: Shaking or tremor causes problems with most or all activities.

132. In comparison with the two weeks BEFORE social distancing, in the last week, shaking or tremor:

(1) Is a little worse

(2) Is much worse

(0) Hasn’t changed

(-1) Is a little better

(-2) Is much better

133. GETTING OUT OF BED, A CAR, OR A DEEP CHAIR

Over the past week, have you usually had trouble getting out of bed, a car seat, or a deep chair?

0: Normal: Not at all (no problems).

1: Slight: I am slow or awkward, but I usually can do it on my first try.

2: Mild: I need more than one try to get up or need occasional help.

3: Moderate: I sometimes need help to get up, but most times I can still do it on my own.

4: Severe: I need help most or all of the time.

134. In comparison with the two weeks BEFORE social distancing, in the last week, trouble getting out of a bed, a car seat or a deep chair:

(1) Is a little worse

(2) Is much worse

(0) Hasn’t changed

(-1) Is a little better

(-2) Is much better

135. WALKING AND BALANCE

Over the past week, have you usually had problems with balance and walking?

0: Normal: Not at all (no problems).

1: Slight: I am slightly slow or may drag a leg. I never use a walking aid.

2: Mild: I occasionally use a walking aid, but I do not need any help from another person.

3: Moderate: I usually use a walking aid (cane, walker) to walk safely without falling. However, I do not usually need the support of another person.

4: Severe: I usually use the support of another person to walk safely without falling.

136. In comparison with the two weeks BEFORE social distancing, in the last week, these problems with balance and walking:

(1) Is a little worse

(2) Is much worse

(0) Hasn’t changed

(-1) Is a little better

(-2) Is much better

137. FREEZING

Over the past week, on your usual day when walking, do you suddenly stop or freeze as if your feet are stuck to the floor?

0: Normal: Not at all (no problems).

1: Slight: I briefly freeze, but I can easily start walking again. I do not need help from someone else or a walking aid (cane or walker) because of freezing.

2: Mild: I freeze and have trouble starting to walk again, but I do not need someone’s help or a walking aid (cane or walker) because of freezing.

3: Moderate: When I freeze I have a lot of trouble starting to walk again and, because of freezing, I sometimes need to use a walking aid or need someone else’s help.

4: Severe: Because of freezing, most or all of the time, I need to use a walking aid or someone’s help.

138. In comparison with the two weeks BEFORE social distancing, in the last week, these sudden stops or feet stuck to the floor:

(1) Is a little worse

(2) Is much worse

(0) Hasn’t changed

(-1) Is a little better

(-2) Is much better
