# Supplementary material for: Multidimensional Factors Can Explain the Clinical Worsening in People With Parkinson's Disease During the COVID-19 Pandemic: A Multicenter Cross-Sectional Trial
Source: Front Neurol. 2021 Jul 30;12:708433. doi: 10.3389/fneur.2021.708433 (PMC8362931; doi:10.3389/fneur.2021.708433)
Supplement: Supplementary file 2 [file Data_Sheet_2.DOCX]

**Supplementary Material II**

**Physical Activity Volume**

Physical activity refers to any bodily movement produced by skeletal muscles that results in expenditure of energy and includes both structured and unstructured activity ([1](#_ENREF_1)). Thus, people with PD answered the following questions: Do you practice any kind of physical activity (question number 64)? What were the duration in minutes of your practice (question number 68)? How many times a week (question number 69)? We computed the physical activity volume from questions number 68 and 69 using the values of two continuous variables, such as session duration and frequency. As previously published ([2](#_ENREF_2)), volume/dosage of physical activity can be computed by multiplying session duration by frequency (times a week).

1. Bangsbo J, Blackwell J, Boraxbekk CJ, Caserotti P, Dela F, Evans AB, et al. Copenhagen Consensus statement 2019: physical activity and ageing. *British journal of sports medicine* (2019) 53(14):856-8. doi: 10.1136/bjsports-2018-100451. PubMed PMID: 30792257; PubMed Central PMCID: PMC6613739.

2. Ludyga S, Gerber M, Puhse U, Looser VN, Kamijo K. Systematic review and meta-analysis investigating moderators of long-term effects of exercise on cognition in healthy individuals. *Nature human behaviour* (2020) 4(6):603-12. doi: 10.1038/s41562-020-0851-8. PubMed PMID: 32231280.
